# Selections

**Published:** 1817-08

**Authors:** 


### PART III.

### SELECTIONS.

—— Factorum est copia nobis.

### FOREIGN WORKS.

### I. PHYSIOLOGY.—*Dissertation on the Circulation of the Blood through the Veins.**

THE author of this very well-written paper sets out with enumerating and briefly controverting the various hypotheses which had previously been advanced in explanation of the causes of the motion of the blood in the veins. These hypotheses rest on the different grounds of elasticity of the venous structure; pulsation of contiguous or adjacent arteries; valvular action; propelling force of the heart and arteries, and contraction of the muscles. The inadequacy of any of these powers singly, and even of all of them conjointly, to produce the phænomena of venous circulation is now

* *Dissertatio de motu sanguinis per Venas; Auctore Josepho Zugenbuhler, Med. Doct.* Mense Januarii, 1815.—*Recueil periodique de la Societé de Medecine de Paris,* Juin, 1815.—We regret that our limits will not allow us to transcribe the whole of this very creditable production. It is clearly and correctly written in the Latin language. EDIT.

so evident, and so generally acknowledged, that we shall not retrace the steps of Dr. Zugenbuhler's refutation. His own views upon the subject are afterward thus exposed:

"My idea is this—The parietes of the right ventricle of the heart are compressed, during the systole, to expel the blood. At the moment of dilatation of the heart, a vacuum is formed; and according to the laws of the pressure of fluids, the blood is driven into the right ventricle. We cannot doubt that, in any receptacle hermetically closed, a vacuum will be formed, upon dilatation of its compressed parietes, and that a neighbouring fluid will rush into it from the pressure of the (external) air. Such a receptacle is the heart. It is filled by the adjacent blood from the influence of the air which surrounds the living body. We therefore place the principle of the venous circulation in the heart. Hitherto, it has been referred to the venous system itself. The action may, indeed, be called the respiration of the blood. It is readily intelligible how, the vena cava being thus emptied, the next portion of blood is, in like manner, raised to that vessel, and thus the whole venous system exhausted as in a syphon."

The following is a condensed view of the *principal arguments* adduced by Dr. Zugenbuhler, *in support of his theory*. The older anatomists were accustomed to inject the different parts and viscera, by placing them beneath the receiver of an air-pump, and subsequently exhausting the air. Hurtful congestions of blood must result, if the venous blood were not returned to the heart with a celerity equal to the action of the arteries. Now the commonly assigned causes of circulation are inadequate to the equable motion of the blood. Not so with the hydraulic operation. The heart constantly absorbs the same portion of blood from the veins as is given out by the arteries. The force which propels the blood from the heart into the arteries, and that which raises it from the veins to the heart are equal; but one acts by propulsion, the other by suction. No one, who knows the impetus with which fluid rushes into a vacuum, will deny that the hydraulic force is proportionate to that of the heart and arteries. Both are exactly proportioned to each other. This theory explains the accelerated motion of the venous blood as it approaches the heart, the deficiency of valves in the vena cava and abdominal veins, rendered needless by the imbibing action of the heart; and the motion of the blood through varicose veins, where the elasticity of the vessel is destroyed: the action of the adjacent arteries is completely inadequate to even a partial propulsion of its contained blood, and muscular pressure would rather operate in inducing than removing the accumulation.*

* We insert the following passage, as evidently bearing upon some doctrines at present warmly contested among us, respecting the physiology of the circulating system:—"Cordis diastole vacuum oritur, et venosus sanguis imbibitur. Cor sanguine repletum irritatur ad reactionem, et systole

Dr. Zugenbuhler's *refutation of supposed objections* is less satisfactory. Unable to follow him through every point of defence, we shall pause upon two or three, merely for the sake of specimen:—The swelling of a vein below a ligature, confessedly a formidable objection, he explains by the impossibility of the formation of any vacuum in the living body, and the certainty that, even if formed, it would instantly be filled by the neighbouring fluid. The circulation of the blood in some insects, where, from deficiency of a heart, no vacuum may be supposed to take place, by the remark that many of the inferior orders of animals possess valves or sacculi instead of heart, by the serpentine contraction of which a species of vacuum may be formed. The muscularity of the vena cava, by the supposition that the presence of this structure is destined to support the elasticity of the vessel; for, were it necessary to the propulsion of the blood, the venous system in general would not be destitute of muscular fibre; and, lastly, the protraction of life, in certain animals, after detachment of the heart, by the assertion that the venous action may, under these circumstances, be kept up for a while by the *auxiliary* causes of circulation; that life, here, should be distinguished from circulation, as, in animals of less complicated structure, the former may survive the latter; and that if circulation do not immediately cease, it may be owing to convulsive contraction of the muscles, for he means not to deny that muscular action is an auxiliary, but merely that it is the principal and essential cause of circulation.

This theory, he adds, if eventually established, would go far to explain several physiological phænomena, particularly the arterial and pulmonary circulation, and the action of the lymphatics. And, in his opinion, the return of the pulmonary blood to the left ventricle admits of readier explanation by a vacuum than the aid of respiration, if the danger attendant on slight congestion of the lungs be considered.

We transcribe the concluding paragraph:—"Respecting the motion of the lymph, no plausible explanation has been advanced by physiologists. No vascular elasticity, no muscular action, or arterial vibration can here operate on the innumerable tubes of the absorbent system, and their tenacious lymph; nor will the theory of capillary tubes solve the difficulty in granulated vessels. Suction, then, caused by the vacuum of the heart, demands our consideration: for by this the thoracic duct pours its contents, drop by drop, into the left subclavian vein, and thus, at a slow but unerring pace, exhausts the lymph from the whole body. The granulated structure of these vessels resembles the valves of veins; hereby congestion is prevented, and one drop after another is uninterruptedly raised. Nor is it without obvious reason that the thoracic duct

---

cruor expellitur. *Durante systole sanguis venosus quiescit*, donec dilatatione novâ cordis in vacuum irruat; et sic rhytmo alternante, vires mechanicæ cordis perennant."—Edit.

empties itself into the left subclavian vein, near the heart; for the left ventricle absorbing the blood more readily from the lungs than the right, may thus exercise a greater power upon the thoracic duct."*

---

### II. PATHOLOGY.—*Case of numerous Hydatids lodged in the Ventricles of the Brain, and firmly connected with the Plexus Choroides.*†

G. R. Pavese, aged about fifty-five, of weakly constitution, ill-fed, for three months subject to intermittent fever, and suffering greatly from mental depression, was attacked on the morning of November 26th, 1797, in the public street, with violent torpor of the lower limbs. Having staggered home, he was instantaneously stricken with a violent pain in the superior and central part of the head; and, making signs for assistance, fell completely senseless to the ground. On being immediately conveyed to the hospital, he was found labouring under the symptoms of asthenic or nervous apoplexy. Internal and external stimulants were applied without effect, and the patient died towards midnight. The body was removed to the anatomical theatre of the University for examination. The cranium being opened, nothing peculiar was observed in the external substance of the brain; but the lateral ventricles, on exposure, were turgid with bloody serum, and two large clusters of hydatids lay along the choroid plexuses, to which they were so closely connected that their detachment could not be accomplished without laceration of the substance of each plexus. Both clusters of hydatids were about two inches long, large, and extended at the inferior extremity, which swam in the bottom of the ventricle, and terminated in an apex with a long cord, bent in various directions.

---

* It is curious that Dr. Zugenbuhler, on the continent, and Dr. Carson, in this island, probably wholly unconnected with, and unknown to, each other, should have been together treading the same path of physiological inquiry, and have arrived at nearly similar results The theory of the Liverpool writer, however, appears to us to be by far the most full and explicit. Nevertheless, we think that Dr. Zugenbuhler's dissertation is well worth the perusal of Dr. Carson, and of all who feel interested in that gentleman's very able researches on the venous circulation. EDIT.

† We translate this case from an Italian work, *on the worms which infect the human body.* It is the production of *Valeriano Luigi Brera;* and entitled, *Lezioni Medico-pratiche sopra i principali vermi del corpo umano vivente e le così dette Malattie verminose.* The whole section is transcribed as a specimen of the Italian Professor's style Of the work itself, we purpose shortly to give an analysis, as it is both valuable and little known in this country. Hydatids, although not commonly occurring, have also been found in the human brain by *Treutler* and *Z[illegible]der.* See the review of *Joerden's* splendid *Entomologie und Helminthologie des menschlichen Koerpers,* in the 8th volume of the *Edinburgh Med, and Surg. Journal.*—EDIT.

By this, they were strongly attached to the septum which anteriorly separates the two ventricles. On removal and accurate examination of this double and regularly disposed mass of hydatids, it appeared that each vesicle constituted an animal of very singular structure.

It consisted of a head, not unlike that of the tænia, and of a vesicle filled with water and beautifully organized. The vesicle was apparently formed by three different membranes: the first external, delicate, transparent, and shining; under which was observed a series of minute circular fibres; and, lastly, a membrane which invested with its villous surface *(superficie velutata)* the interior of the vesicle. Each vesicle, therefore, was but one worm.* The cavity of the vesicle contained nothing but water; and not the slightest trace of any organ subservient to the natural functions could be discerned. The figure of the vesicle was round, oblong, or angular. If the animal were alive, on slight pressure of its neck, the head started out, furnished with piercers and a little mouth, resembling those of the *tænia armata.*

This worm has formerly been found by celebrated physicians, not only in the brain, but in the various parts of the human body.† According to the observations of Koelpin,‡ and Walter,§ the greater part of the hydatids, so called, really belong to this species.||

---

* Hence it has, by Block, *(Traité de la generation des vers des intestins*, &c. ii, Esp. p. 52) been called *solitarius (eremita)* to distinguish it from the *vescicolaris socialis,* which consists of a vesicle filled with from three to four hundred very small worms. The former has been confusedly described by writers under various names. It is the *hydatis animata* of *Peyerus (Miscell. natur curios.* Dec. 1, Ann. vii. Obs. 206); the *ova in porcis* of *Bartholinè (Histor. anat. rarior.* C. ii. Obs. 87, p. 293); the *lumbricus hydropicus* of *Tyson (Phil. Transactions,* Vol. xvii. p. 506); the *hydra hydatula* of *Linnæus (Systema naturæ,* Edit. xii. p. 1320); the *tænia hydatoidea* of *Pallas (Elenchus Zoophytorum,* &c. No. 413); the *tænia vesicularis* of *Goeze, (Versuch einer naturgeschichte,* &c. p. 248.) and the *tænia hydatigena* of Fischer *(Tæniæ hydatigenæ in plexu choroideo inventæ historia,* &c.) and of *Werner (Vermium intestinalium,* &c. p. 66.) *Brera* has given it the "more appropriate designation" of *vermis vescicolaris.*

† In the *brain, Ludwig, de hydrop. cerebr. puer.* Lipsiæ, 1774; *Hufeland, ueber die natur,* &c. *der Skrofelkrankheit,* Jena, 1795; *Weikard, Vermischte medic. schriften.* iv. St.; *Medical Facts and Obs.* London, 1792: In the *liver, Baillie, Morbid Anatomy:* Beneath the *pectoral muscles, Werner, Vermium intestin.* &c. curante Fischer: In *tumours, Hunter, Medical and Chirurg. Transactions,* London, 1793.

‡ *Melanges par la Societé des curieux,* &c. a Berlin, Vol. i.

§ *Block, Op. citat.* p. 54.

|| *Zeder, Erster nachtrag zur naturgeschichte.* Prof. *Walter* assured *Block* that, on opening bodies, he had seen some of the *vermes vescicolares* issue from hydatids. Yet *Werner,* (Op. cit.) having examined the membrane of the hydatids, did not find it organized like that of the *vermis vescicolaris.*

Pallas is inclined to think, that encysted dropsy may be produced by a collection of the *vermis vescicolaris.** 

From accumulated observation it appears, that this worm prefers for its habitation those parts of the body which are most liberally endowed with lymphatic vessels. Attaching itself by the head to their branches, the animal thus fills with the contained lymph the vesicle which constitutes its body. The unciniform protuberance with which the head is furnished, and somewhat resembling a circle of wrinkles, doubtless, serves to fix the papilla, in the centre of the head, against that part of the human body from which the worm absorbs its nutriment.

It is singular, that this worm is only discovered in the deeper recesses of the human frame, and deprived of the slightest communication with the exterior. Hitherto, no trace of its ova has been detected. Are they never developed in the cavity of the lymphatic vessels? †

The *vermis vescicolaris*, just described, essentially differs from that which is found in the liver of the hare and mouse, and in the sheep's brain; notwithstanding considerable resemblance exists between them. In the former, as was before remarked, each vesicle constitutes but one worm; while, in the hydatid of other animals, many small worms are lodged in the same vesicle. The head of the human *vermis vescicolaris* is external to the sac, and connected to it by the medium of the neck; but in that of animals, the little worms are lodged within the parietes of a common vesicle. Finally, the vesicle of the human hydatid appears to constitute the real body of the worm; but the sac of the hydatid of animals serves only the purpose of a common receptacle. The delineations of the *vermis vescicolaris* of the hare and sheep, given by *Goeze*, are very correct, and merit attention, as elucidating the intrinsic and essential difference which exists between it, and the *vermis vescicolaris* of man. *Block* is the only one by whom they have been accurately distinguished.

---

### *Case of Disease of the Colon, terminating in fatal Disorganization; which, during Life, had been mistaken for Aneurism of the Heart* ‡

Dr. Duchateau, after some observations on the fallacy of diagnosis

---

* *Nordische Beytrage.* 1 Band; *de Haen, Ration. medend.* p. 3, vol. ii; *Morand, dans les Memoires de l'Acad. de Paris,* 1772; *Wagler, Lib. de morbo mucos,* Goettingæ, 1762.

† "Hydatids are sometimes real, varices of the lymphatic vessels." *Soemmering, de morbis vasor, absorb. corpor. humani.* Trajecti ad Mœnum, 1795.

‡ *Observation sur un pretendu aneurisme du cœur. Par M. Duchateau, M. D. Bulletin de la Societé Médicalé d'Emulation.* No. IX. Sept. 1815.

in organic affections, narrates an history, of which the following is a correct sketch; and which cannot be too strongly recommended to the notice of every practitioner.—A man, aged 25, of strong constitution and sanguineo-lymphatic temperament, subject to piles, and having quitted the army for a commercial situation in Paris, exhibited the following symptoms, when first visited by Duchateau: Countenance pale; lips discoloured; unnatural corpulence; chest and shoulders raised; respiration tight; and hoarseness. Exposed to great bodily exertion, he frequently came home, covered with perspiration, and breathless. He ate and drank well. Nothing was done. In March, 1808, he was suddenly seized with suffocation, acute and dry cough, violent oppression, bilious vomitings, and syncope. Duchateau found him pale, apparently dying; pulse scarcely perceptible; tongue loaded with a yellow fur. Hence he suspected gastric disorder; but the man, on recovery, complained of intolerable palpitation of the heart, load at the stomach, constriction of the chest, and violent head ache. The pulse was full, hard, irregular, particularly in the left arm; epigastrium tense; parietes of the chest elevated by frightful pulsations, and rendering an obscure sound on percussion. On being questioned respecting the palpitation, the patient said, that it first attacked him three years before, without evident cause; had been greatly aggravated of late; but that he had submitted to no treatment; *as his disease had been pronounced by all the physicians whom he had consulted, and particularly by Corvisart, to be aneurism of the heart.*

By the aid of medicine,* the man recovered his former state; but, aware of his situation, would submit to no restrictions. *Corvisart was again consulted, and confirmed his former diagnosis.* Another year passed without alteration, except that, for the last month, the oppression was aggravated; the cough more dry; palpitations more tumultuous; and walking impeded by a sense of painful anxiety about the præcordial region. On February 21st, 1809, after great exertion in cold weather, this man was seized with general uneasiness, convulsion, syncope, and expectoration of florid blood. Copious vomiting of red blood and black coagula ensued: and among the food, accompanying them, were observed some gland-like and fatty substances, compact, and of an ash-white colour, so as to resemble the pulmonary tubercle. They were washed, and preserved in vinegar.

Blood continued to be discharged both by vomiting and stool, and was always accompanied with portions of the substance just described. Convulsive spasm, slight delirium, unaccompanied by severe suffering, weakness and concentration of the pulse, ex-

---

* The remedies, prescribed by Duchateau, were successive applications of leeches to the throat, anus, and thorax; antispasmodics; purgatives and laxative injections; chicken broth mixed with orange-flower water, and syrup of violets; and lemonade.

tinction of the voice, insatiable thirst, and dryness of the tongue preceded death, which took place on the fourth day.

*Dissection on the 28th of February.* All the *thoracic organs were sound;* as were the stomach, liver, gall-bladder, spleen, and pancreas. On opening the intestinal tube from the pylorus, its duodenal and jejunal portions appeared healthy; but the ileum, for about two feet from its junction with the colon, was livid and gangrenous throughout. The colon itself was distended and blackish; its whole right portion sphacelated, and perforated to the extent of a crown-piece. The membranes were so much altered as to break beneath the finger. The whole adherent portion of omentum, which had escaped destruction, was of a dark-grey colour; the detached part had escaped into the intestine, and had followed the route of the fluids evacuated by stool and vomiting. The intestine, opened throughout, presented internally the same black colour; and the mucous membrane, the want of consistence, consequent on gangrene.

The *Reflections* of Dr. Duchateau we shall pass over, with merely noticing his opinions, that, in this case, part of the omentum had, at first, been the seat of an insidious chronic affection,* and that, contracting adhesions to the ascending colon, it had thus excited passive inflammation of the intestine, until the latter, perforated by the gangrene, had given passage to the portions of omentum, which were subsequently discharged by the mouth and rectum. Lastly, that the palpitations resulted from a lesion of some nervous branches of the mesenteric plexus sympathising with the nerves of the heart.

---

### III. PRACTICE (*Medical*)—*Case of periodical Salivation and Head-ache successfully treated.*†

A journeyman tailor, aged 26, of strong constitution and born of healthy parents, had suffered from his eighth year, at which time he went through the small-pox regularly, very frequent and

---

* We have, ourselves, no doubt but that the abdomen was, in this case, the seat of the primary disease, and that the physiological lesion of the heart and lungs was purely sympathetic. Asthma itself is frequently known to originate from epigastric derangement: and in persons of irritable heart, any irritation of the abdominal viscera, or cause accelerating the stream of blood in its transit through the venæ cavæ and lungs, will throw the heart into a state of violent and irregular action, very difficultly distinguishable from organic disease. Hence the practitioner cannot be too circumspect in delivering an opinion on such cases. At this moment, there stands before our window, a healthy man, whom, many years ago, we gave up, as the victim of active dilatation of the heart, and who was subsequently cured, to our astonishment, by the aloes, spirit of ammonia, and bitters, of a sagacious empiric. —~~Edit.~~ [illegible]

† *Periodischer Speichelfluss und Kopfschmerz beobachtet und beschrieben von Dr. Woyde.* Journal der Practischen Heilkunde, &c. Nov. 1815.

severe head-aches, invariably complicated with an increased flow of saliva, and often with vomiting. The paroxysms, at first, came on but rarely; and, for a long time, the patient was completely without them. Afterwards, however, they grew more frequent, and now occurred three or four times a week. The pain was commonly strongest in the frontal region, and fixed itself, during the paroxysm, in one or other half of the head. The patient had never been affected with hæmorrhoidal complaints or rheumatism.

He was admitted on February the 2d. His cachectic countenance and remarkably slow pulse excited particular attention. His appetite was but small; his thirst and bowels natural. In the intervals between the paroxysms, the patient felt himself nearly well. The disease was regarded, in its intrinsic nature, as a periodical nervous rheumatism; and hereupon the indications (of cure) were founded. A powder, composed of *calomel*, *sulphuret of antimony*, *gum guaiacum*, and *extract of henbane*, was directed evening and morning, and two large spoonfuls of a decoction of *colombo* and *valerian roots*, with *sulphuric æther*, three times a day, but without the desired effect. The paroxysms of head-ache decreased in frequency, but not in strength; and the attendant salivation was considerably aggravated. A *perpetual blister* was now laid on the neck; and to powders of *extract of aconite* was added a decoction of *guaiacum shavings*, with *æthereal tincture of valerian*. But the disease took no more favourable turn. The paroxysms invariably came on, as before, every third or fourth day; commonly continued through the whole day with most violent salivation; and, from their severity, incapacitated the patient for all business. In the intervals, he still found himself nearly well. Hence it was determined to treat the disease as a periodical nervous affection, with an increased secretion of saliva, analogous, in character, to periodical diarrhœa, or hæmorrhoidal flux; and to make its periodicity the main object of the cure. The previously employed remedies were therefore discontinued, and the patient was desired to swallow, every two hours, a tea-spoonful of an electuary composed of *cinchona* and *valerian*. Thus, till the 11th of March, he was completely free from his paroxysms: yet they afterwards returned, although weaker, on the third, fourth, and eighth days. The salivation was somewhat diminished, and the man could now work during the paroxysms, and transact his ordinary business. On the 18th, a drachm of *radix serpentariæ* was added to the electuary. From the 22d of March to the 6th of April, the paroxysms returned more violently, and observed a tertian type. Besides the *cinchona*, *serpentaria*, and *valerian*, which were henceforth given in the form of powder, the patient got, on April the 6th, half a grain of *radix belladonnæ*, night and morning. Hence, from that day to the 15th, he experienced only two very slight paroxysms; and from then to the 18th, he remained perfectly free. Upon discontinuing the *belladonna*, however, on April 28th, the paroxysms recurred: but the repetition of it to two grains daily, caused them to return but very seldom, and without salivation. Afterwards, half a drachm of the *flores salis ammon. mart.* (ferrum ammoniatum) was added to the powders, and the paroxysms wholly disappeared. From the 5th of June to the 5th of August, he took nothing, and had only twice paroxysms very mildly. He got, from that time, completely well.

### Practice *(Surgical.) — Observations on certain Gun-shot Wounds, accompanied by peculiar Circumstances.**

The celebrated Baron Larrey is the author of this communication. It is well known by army surgeons, that the existence of one wound only upon a limb struck by a musket-shot, is no certain proof that the ball remains in the substance of the part. Many causes may operate its escape by the same track, whatever be the extent or direction of the wound : but on the nature of the causes, and their mode of operation, authors are not agreed; some having thought, that the expulsion of the ball was caused by its rebounding from the tendons, fasciæ, or bones of the limb; others, that it was effected by muscular contraction. But animal physics and experience alike demonstrate the error of these opinions : for, first, the ball, destitute of elasticity, cannot rebound from the parts upon which it strikes, whatever be their density, and however feeble the shock of the projectile; and this, moreover, is partly or wholly flattened, or cut into two or more pieces, according to the resistance with which it meets; and the parts stricken undergo a relative alteration: and, secondly, the moving fibre, when struck by the ball, is instantly deprived of its contractility, and, however slight be the shock, contused and lacerated, and the ball lost in the substance of the muscle; hence the action of the moving power on the foreign body is nearly or altogether annihilated. How then is the repulsion of the ball through the wound, which it has made on entrance, to be explained?

If the wound, inflicted on the soft parts, be not very deep, the projectile may be repelled through this track by some portion of clothes which has been driven in before it. In fact, when the track is short, the *finger-glove*, containing the ball, is frequently brought to the exterior by the motions, or change of posture, of the patient, and the foreign body either remains in his clothes, or falls to the ground. But when the wound is deep, the orifice of the integuments contracts, from their organic properties, and enlargement of the wound to extract the ball becomes necessary. This opinion is confirmed by the following Cases, in which "Nature, as it were, has been surprized in the act."

Case 1. A grenadier received, in the battle of Craone, 1814, a musket-shot in the left thigh. He walked to the *ambulance*

---

* *Notice sur certaines Plaies d'Armes a Feu, accompagnées de Circonstances particulieres; par M. le Docteur Baron Larrey.* Bulletin de la Faculté de Médecine de Paris, 1816, No. 2.

stooping, the thigh and leg bent, and severe dragging pains felt in the wound. On examination, a portion of shirt was seen deeply engaged in a round and scarcely perceptible wound in the internal and upper part of the limb: it seemed to burrow in the cellular structure which separates the tendons of the *triceps adductor* from the *sartorius*, and towards the trochanter minor. Useless efforts were made to extract the piece of linen; and Larrey being summoned, after dilating the superior and inferior angles of the wound, removed it by gradual traction, and found, contained in it, a large ball and a portion of the stuff of the breeches. The ball was flattened, and one of its surfaces slightly fretted, as though it had encountered the summit of the trochanter. In fact, a female catheter, introduced into the wound, passed beyond this eminence towards the *linea aspera*. Treated as a simple one, the wound was soon cured.

Case 2. At the disastrous battle of *Mont St. Jean* (Waterloo), a young soldier of the line received a shot, which, after traversing the waistband of the breeches, had penetrated a little above the right spermatic vessels. It had carried before it a portion of shirt, and travelled under the integuments and cord to the external side of the testicle; so that the linen formed a *finger-glove*, as in the last case, at the bottom of which was the ball. The man's body was bent from the pain. On dilating the wound, the foreign substances were readily extracted; and the cure was completed without injury to the testicle.

Case 3. A soldier came to the military hospital at *Gros-Caillou*, in January 1816, to be cured of a fistulous wound on the right side of the abdomen, two fingers' breadth from the margin of the false ribs. The wound was small, round, and directed obliquely from right to left and inwards. A probe penetrated in this direction into the abdominal cavity, towards the transverse colon and anterior surface of the stomach. By the contact of the instrument on these organs, singular nervous paroxysms were instantly determined: at first, a sensation of cold, with compressive pain; afterwards, spasmodic contraction of the whole abdomen, with yawning and stretching; inordinate loquacity, and a kind of somnambulism, which might be protracted by following the thread of the patient's discourse. In about thirty minutes these symptoms disappeared, and he relapsed into the state of nostalgia and hypochondriasm, in which he had been from the receipt of his wound. An orange-coloured fluid was constantly discharged from the orifice, presenting all the characters of peritoneal serum. It appeared, on inquiry, that the shot, which he had received on the 10th of April 1815, in Catalonia, had driven before it a portion of shirt into the abdominal cavity. The man immediately fell; and one of his comrades, unable to extract the piece of linen, cut it off on a level with the wound. A Spanish surgeon, to whose care he was confided, in the hospital of *Figuieres*, contented himself with simply dressing the wound. Inflammatory symptoms were soon developed; the abdomen swelled, and the patient voided a large quantity of blood by stool, after experiencing violent colics and propensity to vomit. By the use of diluents and emollient poultices, these symptoms were, in a few days, subdued, and an abundant suppuration was established. After three months, the surgeon, directed by a piece of linen which had reappeared in the bottom of the wound, dilated its borders, and extracted the foreign body. The linen formed a sac of four fingers' breadth long, and in the bottom of it was the ball. From this moment the patient recovered, and was subsequently evacuated on the French hospitals.

It seems probable, that the ball, enveloped in the linen, after having overcome the firm resistance opposed to it by the muscular and fascial parietes of the abdomen, had struck the corresponding portion of the transverse colon, through the intervening omentum, and ruptured the internal vascular membrane of the intestine, whence the intestinal hæmorrhage; and that the primary inflammatory symptoms were consequent on the perforation of the abdominal parietes, and commotion of the nervous branches of the solar plexus. The hypochondriasm and other spasmodic symptoms, felt periodically or on the introduction of the probe into the wound, are referred by Larrey to "exaltation of the pneumo-gastric and splanchnic nerves, anastomosing with the semilunar ganglia, from which the solar plexus originates."

The periodical attacks were much relieved by topical employment of oily and camphorated embrocations, and the internal use of valerian and other antispasmodics; but serum continued to flow from the fistulous orifice.

What then was to be done? Was the wound again to be dilated, with a view of procuring adhesion and perfect cicatrix? To this procedure the objections are, the danger of obstinate hæmorrhage from division of the arteries surrounding the wound, and of extravasation into the abdominal cavity; and the possibility that inflammation of the peritoneum, or its adhesion to the contained viscera, might result from exposure of the membrane to the contact of air. Or should the disease be abandoned to the efforts of Nature? In favour of this opinion, Larrey relates the case of a lady, of Toulon, who, in 1796, underwent the operation of paracentesis, *by incision*, for peritonæal dropsy. The wound, at first fistulous, spontaneously closed in about a year; and, at the period of the French expedition to Egypt, in 1798, the lady's health was perfectly restored. Larrey thinks that the process of adhesion might be accelerated by touching the internal borders of the wound with caustic.

**Practice** *(Obstetrical).—Observations on a particular Species of Hæmorrhage, which sometimes succeeds Delivery.**

Dr. Lobstein, on September 10th, 1804, attended a woman at the Strasburgh hospital in her first labour. Immediately after delivery of the child, hæmorrhage, to no very alarming extent, came on. Suspecting separation of the placenta, he proceeded to extract it. Though not yet detached, its removal was readily effected; and he felt the uterus contract upon his hand: but the hæmorrhage continued. On introduction of the hand again into the vagina, the uterus was found perfectly contracted. Cold applications were used to the abdomen, but the flow of blood was incessant. On separating the labia, a triangular excrescence, resembling a *caruncula myrtiformis*, was discovered at the posterior inferior part of the vagina, somewhat above the *fossa navicularis*. From the summit of this, as from a vein in the arm, a jet of blood sprang. A ligature of waxed thread being applied round its base, the hæmorrhage instantly ceased; and, two days afterwards, the thread came away from the vagina; but what had become of the excrescence which it had tied was not ascertained.

On June 2d, 1805, Dr. Lobstein was summoned to a woman just delivered in the hospital of her first child. He learned, on arrival, that the delivery had been natural; but that, shortly after the expulsion of the placenta, the abdomen had begun to swell, and the woman to complain of great pain in the vagina. The midwife, on examination, thought that she distinguised an inversion of the womb, and hence had sent for the doctor. On introduction of his hand into the vagina, he discovered at its superior extremity a firm tense painful tumour, exactly closing it and preventing farther progress. Unable to make out the uterine orifice, he, at first, thought that the fundus of the womb had descended into, and become strangulated by it; and hence, that the case was the incomplete inversion of the organ described by Baudelocque. But the presence of the hard and globular fundus uteri, near the umbilicus, disproved this notion, and induced him to examine more minutely the connections, relations, and boundary of the tumour. It arose from the posterior wall of the vagina, and the finger might be passed along all the rest of its circumference. Tracing the anterior paries of the vagina, he penetrated into the uterine cavity, filled with coagulated blood. It was now easy to evacuate this blood, to provoke the contractions of the uterus, and examine afresh the nature of the disease. Thus, it proved, that below the uterine orifice a flap of membrane perfectly resembling that of the first case, but larger, had been detached from the posterior wall of the vagina,

---

* *Notice sur une espèce particulière d'Hémorrhagie, qui succède quelquefois a l'accouchement. Par J. F. Lobstein.* Bulletin de la Société Médicale d'Emulation, Jan. et Fev. 1816.

and opposing, like a valve, the issue of the blood from the uterus, had formed a convex tumour, comparable to that which the valves of the aorta and pulmonary artery present towards the ventricles of the heart, when the vessels have been injected in a retrograde direction. The vagina was not perforated, so as to communicate with the rectum or abdominal cavity. The woman's recovery was favourable, except that she was teazed with a cough of long standing. Two years afterwards, she died in the hospital, of phthisis pulmonalis; and, on dissection, a large cicatrix was found in the middle of the posterior paries of the vagina.

Hence, it appears, that considerable hæmorrhage may arise from the rupture of a portion of the internal membrane of the vagina, and its separation from the external. In pregnancy, the vessels of this part dilate like those of the uterus; and the veins especially, acquiring great size, bleed profusely on rupture. This happened in the first case. In the second, the separated membrane was probably not the sole source of the hæmorrhage, or the latter would have continued after the evacuation of the coagula which had arisen from retension of the blood by the valve-like membrane. This rupture, again, although more considerable than in the first case, had not implicated vessels equally large, which are less numerous here than in the inferior part of the vagina, where the venous plexuses are so large that they may even become varicose. The rupture, in both cases, had taken place on the posterior surface of the vagina; and was, doubtless, caused by the passage of the forehead and face of the child along the column of transverse rugæ which this surface presents, and which will obviously be more prominent in a first than in subsequent labours. The two membranes of the vagina, it may be observed, although inseparably united in the natural state, become readily separable, from the laxity of their intervening cellular structure, and other changes which take place during pregnancy. Dr. Lobstein has even seen, on the body of a woman whose perinæum had given way in parturition, the internal membrane ruptured far into the vagina without the slightest injury of the external.

The species of vaginal hæmorrhage, just noticed, is not mentioned by authors. The accident noted by Professor Boer, of Vienna,* under the title of *a yet undescribed species of hæmorrhage which happens to parturient women*, though somewhat analogous, differs, in many respects, from these cases. It consists of complete rupture of the vaginal parietes, with hæmorrhage, and extravasation of blood into the cellular membrane of the pelvis, and even of the labia and buttocks, followed by inflammation and suppuration. In the cases recorded by Lobstein, the internal membrane of the vagina exclusively is ruptured and separated; an accident much less formidable than that which is described by the Vienna Professor: two of the four subjects, in whom it had been

---

* *Abhand. und Versuch. geburts*, &c. Vienna, 1802.

observed by him, having fallen victims to it. The former species of hæmorrhage can only prove fatal from error or neglect, unless it happen in a feeble and exhausted subject whom the slightest accident may destroy, as in the following case, recently observed by Dr. Lobstein.

A weakly, delicate, cachectic female, aged 27, who had suffered from quartan fever during the two last months of pregnancy, was attended by him in her first labour. The process went on favourably till the child's head pressed on the perinæum. A slight hæmorrhage then arose; but again ceased as the head advanced. After the birth of the child, which was small and weakly, though at the full time, the placenta was immediately removed, under the idea that its partial separation had caused the hæmorrhage; but the discharge still continued, even after contraction of the uterus had been induced. The vagina was then plugged, and the bleeding ceased. Yet faintness, discolouration of the countenance, extinction of the pulse, and coldness of the extremities came on; and the woman sank, after slight convulsion, to the utter astonishment and dismay of all around. *On Dissection*, nothing, capable of explaining the woman's sudden death, was discovered in the abdominal or thoracic cavities. The liver was somewhat larger, and the spleen more soft and flaccid, than common. The uterus was sufficiently contracted; its vessels, those of the pelvis, and all the abdominal veins, filled with blood. The vagina was without lesion; but, on minute inspection of the entrance of the vulva, a somewhat large space was found at its lateral and posterior part, where, by abrasion of the mucous membrane, the *plexus retiformis* had been exposed, so that its open cells might be inflated by the blow-pipe. Hence the hæmorrhage had proceeded. It first appeared when the child's head was pressing on the perinæum, and was caused by erosion of the mucous membrane, here very delicate. It ceased during compression of the plexus by the head, and recurred when that pressure was removed. Its source could not be discovered during life; for it was difficult of detection on the dead body. And, even had such an unheard of occurrence in a natural labour, been ascertained, what more could have been done to arrest the hæmorrhage, than was done? The hæmorrhage, if not the sole cause of death, doubtless, contributed essentially to that event, in a delicate and relaxed subject, by inducing direct debility and exhaustion. A case, nearly similar, occurred, some years since, to a friend of Dr. Lobstein. A delicate unhealthy woman sank, from trifling hæmorrhage, after a favourable delivery; and on dissection, no morbid appearance of the internal organs or denudation of the vaginal plexus, was discovered to explain the cause of dissolution.*

---

* We have no doubt that, in these melancholy cases, the fatal effects of the hæmorrhage are frequently determined or accelerated by the influence of terror, apprehension, grief, or other depressing passion, on the woman's mind. EDITORS.

Thus, concludes Dr. Lobstein, vaginal hæmorrhagies occur, which, though less formidable than those of the uterus, demand the attention of accoucheurs, as they may become dangerous, from ignorance or neglect.

### PART IV.

### MEDICAL MISCELLANIES.

### THE PORTE FEUILLE, No. VII.

OR,

DEPOT FOR DETACHED FACTS AND INTERESTING PHÆNOMENA OCCURRING IN THE PRACTICE OF THE FRIENDS OF THE MEDICO-CHIRURGICAL JOURNAL.

"Ore trahit quodcunque potest atque addit acervo."

### *Aneurism of the Arteria Innominata.*

